# Reduced serum cortisol, IGF-1, GLP-1, and T3 levels in medication-free children and adolescents with obsessive–compulsive disorder: a case–control study

**DOI:** 10.3389/fpsyt.2026.1831156

**Published:** 2026-06-03

**Authors:** Fatma Coşkun, Hasibe Ağır, Rukiye Esra Ildız, İbrahim Kılınç, Necati Uzun, Emine Göktaş, Ahmet Osman Kılıç

**Affiliations:** 1 Department of Child and Adolescent Psychiatry, Faculty of Medicine, Necmettin Erbakan University, Konya, Türkiye; 2 Department of Biochemistry, Faculty of Medicine, Necmettin Erbakan University, Konya, Türkiye; 3 Department of Medical Genetics, Faculty of Medicine, Necmettin Erbakan University, Konya, Türkiye; 4 Department of Pediatrics, Faculty of Medicine, Necmettin Erbakan University, Konya, Türkiye

**Keywords:** adrenocorticotropic hormone, cortisol, glucagon-like peptide-1, growth hormone, insulin-like growth factor-1, obsessive–compulsive disorder, relaxin-3, triiodothyronine

## Abstract

**Introduction:**

Obsessive–compulsive disorder (OCD) frequently emerges during childhood and adolescence and has been associated with alterations in multiple neurobiological systems. Neuroendocrine pathways involved in stress regulation, neurodevelopment, metabolic signaling, and thyroid function interact closely to influence brain circuits implicated in OCD. However, studies simultaneously examining several neuroendocrine parameters in pediatric OCD remain limited.

**Methods:**

This case–control study included 52 medication-free children and adolescents with OCD and 42 healthy controls aged 8–17 years. Psychiatric diagnoses were established using the Schedule for Affective Disorders and Schizophrenia for School-Age Children—Present and Lifetime Version (K-SADS-PL) according to DSM-5 criteria. Obsessive–compulsive symptom severity was assessed using the Children’s Yale–Brown Obsessive Compulsive Scale (CY-BOCS). Anxiety and depressive symptoms were evaluated using the Screen for Child Anxiety Related Emotional Disorders (SCARED) and the Children’s Depression Inventory (CDI), respectively. Serum levels of adrenocorticotropic hormone (ACTH), cortisol, growth hormone (GH), Insulin-like growth factor-1 (IGF-1), Glucagon-like peptide-1 (GLP-1), relaxin-3 (RLN-3), triiodothyronine (T3), and thyroid-stimulating hormone (TSH) were measured using ELISA. Group differences were examined using covariance-based models controlling for age, sex, BMI percentile, anxiety (SCARED), and depressive symptoms (CDI).

**Results:**

Initial analyses showed that children and adolescents with OCD had significantly lower serum levels of cortisol, IGF-1, GLP-1, and T3 compared with healthy controls. These differences remained significant after controlling for age, sex, BMI percentile, and the SCARED and CDI total score, with adjusted analyses showing lower levels of cortisol (p = 0.003), IGF-1 (p = 0.013), GLP-1 (p = 0.017), and T3 (p = 0.015) in the OCD group. Correlation analyses did not reveal significant associations between biochemical parameters and OCD symptom severity after correction for multiple comparisons.

**Discussion:**

These findings suggest that pediatric OCD may be associated with alterations in several neuroendocrine pathways rather than isolated changes within a single hormonal system. Further longitudinal studies are needed to better clarify the biological basis and clinical significance of these findings.

## 1 Introduction

Obsessive–compulsive disorder (OCD) is a psychiatric condition characterized by recurrent intrusive thoughts and repetitive behaviors that individuals feel driven to perform in order to reduce anxiety or distress, and it frequently emerges during childhood or adolescence (1). Although the exact mechanisms underlying OCD remain incompletely understood, current research suggests that its development involves a complex interaction of genetic vulnerability, environmental influences, and neurobiological alterations (2–5). In particular, abnormalities in cortico-striatal-thalamo-cortical circuits, along with dysregulation of neurotransmitter and neuroendocrine systems involved in stress and behavioral regulation, have been implicated in the pathophysiology of the disorder (6–8). Stress-related mechanisms have been suggested to play an important role in both the onset and exacerbation of OCD symptoms (9). Previous studies have suggested that dysregulation of hormonal and neuropeptide systems involved in stress response, metabolic regulation, and neurodevelopment may contribute to the biological mechanisms underlying OCD (10–12). However, most neurobiological studies on OCD have primarily focused on adult populations, and studies examining pediatric samples remain limited.

The hypothalamic–pituitary–adrenal (HPA) axis represents one of the principal neuroendocrine systems involved in the regulation of the stress response (9). Because stress is considered an important factor in the onset or exacerbation of OCD symptoms, several studies have investigated potential alterations in HPA axis activity in OCD (8, 9, 12–17). Most studies examining HPA axis activity in OCD have primarily focused on cortisol secretion, although a smaller number of studies have also assessed adrenocorticotropic hormone (ACTH) levels. Some findings suggest increased basal cortisol levels or altered stress responses, particularly in adult samples (9, 13, 15, 16). In contrast, data from pediatric populations remain limited. Studies assessing both ACTH and cortisol in children and adolescents with OCD have reported inconsistent results, with some indicating elevated levels of these hormones (17), while others have observed increased ACTH concentrations without significant differences in cortisol levels (8). These findings highlight the need for further research to better understand the role of HPA axis activity in OCD, particularly in pediatric populations.

In addition to classical HPA axis hormones such as ACTH and cortisol, several neuropeptide systems have also been suggested to modulate stress-related neuroendocrine responses, including the relaxin-3 (RLN-3) signaling system (18). RLN-3, also known as insulin-like peptide-7 (INSL7), is a neuropeptide predominantly expressed in the brainstem and has been implicated in the regulation of stress-related neuroendocrine responses (19). Through its receptor RXFP3, RLN-3 signaling influences limbic and hypothalamic circuits and may modulate hypothalamic Corticotropin-Releasing Hormone (CRH) neurons involved in the regulation of the HPA axis (18, 20). Experimental findings suggest that this neuropeptide system may influence emotional regulation and anxiety-related processes (19, 20). A recent study reported altered serum RLN-3 levels in children with autism spectrum disorder, suggesting a possible role of this neuropeptide in social behavior and appetite regulation (21). However, to our knowledge, no studies have yet examined the role of RLN-3 in OCD, highlighting the need for further research. Therefore, investigating RLN-3 alongside classical stress-related hormones may provide additional insight into the neuroendocrine mechanisms underlying OCD.

Glucagon-like peptide-1 (GLP-1) is a peptide hormone primarily involved in glucose metabolism and appetite regulation (22), but several studies suggest that it also exerts effects within the central nervous system. GLP-1 receptors are expressed in brain regions implicated in emotional regulation, reward processing, and stress responses, suggesting a potential role for GLP-1 signaling in behavior and psychiatric symptoms (23–25). Recent studies have further indicated that GLP-1-related pathways may be associated with several psychiatric and neurodevelopmental conditions, including OCD (26). In addition, a recent case report described reduced compulsive food-related thoughts and repetitive behaviors following GLP-1 analog liraglutide treatment in an adolescent with autism, further suggesting that GLP-1-related pathways may be relevant to repetitive and compulsive symptom domains (27). Nevertheless, studies directly investigating GLP-1 signaling in OCD remain scarce, highlighting the need for further research in this area.

Hormonal pathways involved in growth and neurodevelopment may also play an important role in brain function and behavior (28). Insulin-like growth factor-1 (IGF-1), a key component of the somatotropic axis regulated by growth hormone (GH), is widely recognized for its involvement in neuronal growth, neurogenesis, and synaptic plasticity (28–32). IGF-1 can cross the blood–brain barrier and modulate several neurotransmitter systems implicated in psychiatric disorders, including serotonergic, dopaminergic, and noradrenergic signaling (33–35). In addition to these neurotrophic effects, the GH–IGF-1 axis may interact with stress-related neuroendocrine pathways, and IGF-1 signaling has been suggested to represent an anabolic counter-regulatory response to stress-related HPA axis activation (36). Despite this biological relevance, studies examining the role of the GH–IGF-1 axis in OCD remain limited (37–39). Moreover, research focusing on pediatric populations remains particularly limited, highlighting the need for further studies investigating the potential involvement of the GH–IGF-1 axis in children and adolescents with OCD.

Thyroid hormones represent another important neuroendocrine system involved in brain development and the regulation of neuronal activity in the central nervous system (40). Their receptors are widely distributed in brain regions involved in emotional and cognitive processing, including the hippocampus and amygdala (40, 41). In addition, interactions between the hypothalamic–pituitary–thyroid (HPT) axis and stress-related neuroendocrine pathways, including the HPA axis, have been suggested (42). Experimental and clinical studies suggest that thyroid hormones may influence several neurotransmitter systems, such as serotonergic, dopaminergic, and noradrenergic pathways, which are implicated in psychiatric disorders (11, 41, 43). However, studies examining thyroid-related hormones, such as thyroid-stimulating hormone (TSH) and triiodothyronine (T3), in OCD remain limited (43, 44), and further research is needed.

These neuroendocrine systems do not function independently but interact through complex regulatory networks that influence stress reactivity, neurodevelopment, metabolic regulation, and emotional processing (45–47). Many of these systems are closely linked to stress-related regulatory mechanisms, which are considered to play an important role in the onset and course of OCD. Dysregulation across these interconnected pathways may contribute to the biological mechanisms underlying OCD. However, most previous studies have focused on individual hormonal systems rather than examining multiple neuroendocrine pathways simultaneously.

Taken together, an increasing number of studies suggests that multiple neuroendocrine pathways may contribute to the biological mechanisms underlying OCD. However, studies simultaneously examining stress-related, metabolic, neurodevelopmental, and thyroid-related hormonal systems in OCD remain scarce, particularly in pediatric populations. Moreover, to the best of our knowledge, the potential role of GLP-1, RLN-3, and IGF-1 in pediatric OCD has not yet been investigated. Therefore, the present case–control study aimed to investigate multiple neuroendocrine markers reflecting interconnected regulatory systems related to stress regulation, neurodevelopment, metabolic signaling, and thyroid function in medication-free children and adolescents with OCD compared with healthy controls.

## 2 Materials and methods

### 2.1 Participants and procedure

Participants with OCD aged 8–17 years were recruited from the Child and Adolescent Psychiatry Outpatient Clinic of Faculty of Medicine at Necmettin Erbakan University. Inclusion criteria for the OCD group included meeting DSM-5 diagnostic criteria for obsessive–compulsive disorder based on the K-SADS-PL interview and being medication-free for at least six months prior to participation. Exclusion criteria for the OCD group included the presence of any psychiatric disorder other than OCD, including major depressive disorder, anxiety disorders, specific phobia, bipolar disorder, schizophrenia, intellectual disability, autism spectrum disorder, attention deficit and hyperactivity disorder, social communication disorder, or tic disorders. Additional exclusion criteria were the presence of any major medical condition (e.g., renal failure, diabetes mellitus, or cancer), neurological disorder (e.g., epilepsy or cerebral palsy), allergic disease, active infectious illness within the past month, and clinical suspicion of pediatric acute-onset neuropsychiatric syndrome (PANS) or pediatric autoimmune neuropsychiatric disorders associated with streptococcal infections (PANDAS). The control group consisted of healthy children and adolescents aged 8–17 years who presented to the pediatric outpatient clinics of the same institution for routine health check-ups. Inclusion criteria for the control group included the absence of any current or lifetime psychiatric disorder according to the clinical evaluation and K-SADS-PL interview. Exclusion criteria for the control group included the presence of any psychiatric disorder, major medical condition, neurological disorder, allergic disease, active infectious illness within the past month, and clinical suspicion of PANS or PANDAS and any history of psychotropic medication use. This study was approved by the Ethics Committee of Necmettin Erbakan University (Approval date: July 21, 2023; Decision No: 2023/4433; Application ID: 15059). All procedures were conducted in accordance with the principles of the Declaration of Helsinki. Prior to participation, the study procedures were explained to both the children and their parents. Written informed consent was obtained from the parents or legal guardians, and verbal assent was obtained from the children prior to participation.

### 2.2 Diagnostic and symptom assessment

All participants in both the OCD and control groups underwent a comprehensive psychiatric evaluation conducted by a trained child and adolescent psychiatrist. Diagnostic assessment was performed using the Schedule for Affective Disorders and Schizophrenia for School-Age Children—Present and Lifetime Version (K-SADS-PL), and the diagnosis of OCD was established according to the Diagnostic and Statistical Manual of Mental Disorders, Fifth Edition (DSM-5) criteria (48). In addition to the psychiatric evaluation, anthropometric measurements were obtained. Height and weight of all participants were recorded at the time of blood sampling, and body mass index (BMI) percentiles were calculated for each child according to age and sex. OCD symptom severity, depressive symptoms, and anxiety levels were further evaluated using standardized psychometric instruments.

#### 2.2.1 Schedule for affective disorders and schizophrenia for school-age children— present and lifetime version (K-SADS-PL)

The K-SADS-PL is a semi-structured diagnostic interview used to assess current and lifetime psychiatric disorders in children and adolescents (48). In the present study, it was administered by a trained child and adolescent psychiatrist to confirm psychiatric diagnoses and to screen for comorbid conditions. The Turkish version of the K-SADS-PL has been shown to have satisfactory validity and reliability in child and adolescent populations (48).

#### 2.2.2 Children’s yale–brown obsessive Compulsive Scale

The severity of obsessive–compulsive symptoms was assessed using the Children’s Yale–Brown Obsessive Compulsive Scale (CY-BOCS), a semi-structured clinician-rated instrument widely used to evaluate OCD symptom severity in children and adolescents (49). In addition to the total severity score, the distribution of OCD symptom dimensions was also determined based on the CY-BOCS symptom checklist and clinical interview. The Turkish version of the CY-BOCS has demonstrated good reliability and validity (49).

#### 2.2.3 Children’s depression inventory

Depressive symptoms were evaluated using the Children’s Depression Inventory (CDI), a self-report scale designed to assess depressive symptoms in children and adolescents (50). For the Turkish version of the scale, a cut-off score of 19 has been recommended to indicate possible depression. The Turkish version of the CDI has demonstrated adequate validity and reliability (50).

#### 2.2.4 Screen for child anxiety related emotional disorders

Anxiety symptoms were assessed using the Screen for Child Anxiety Related Emotional Disorders (SCARED), a self-report questionnaire used to evaluate anxiety symptoms in children and adolescents (51). Although a specific cut-off score for the Turkish version has not been clearly established, previous studies have reported acceptable validity and reliability for the Turkish adaptation of the scale (51).

### 2.3 Collection of blood samples

Venous blood samples were collected between 8:00 AM and 10:00 AM after an overnight fast using sterile vacuum tubes containing a clot activator. Serum was separated by centrifugation at 1000 × g for 10 minutes at 4 °C (Hettich Rotina 46R centrifuge, Hettich Zentrifugen, Tuttlingen, Germany). The serum samples were aliquoted and stored at -80 °C (New Brunswick U570 freezer, New Brunswick Scientific, New Jersey, USA) until analysis. Serum concentrations of ACTH, cortisol, GH, GLP1, IGF1, RLN-3, T3, and TSH were quantified using commercially available enzyme-linked immunosorbent assay (ELISA) kits (YL Biont Co., Shanghai, China) according to the manufacturer’s protocols. The following kits were used: ACTH (catalog no. YLA1245HU), Cortisol (catalog no. YLA0169HU), GH (catalog no. YLA1703HU), GLP1 (catalog no. YLA0211HU), IGF1 (catalog no. YLA0665HU), RLN-3 (catalog no. YLA4309HU), T3 (catalog no. YLA1497HU), and TSH (catalog no. YLA1712HU). All ELISA procedures were performed using a BioTek ELx50 automated microplate washer (BioTek Instruments, Winooski, Vermont, USA), and absorbance was measured at 450 nm using a BioRad xMark microplate reader (Bio-Rad Laboratories, Hercules, California, USA). The analytical sensitivities (minimum detectable concentrations) were 2.38 pg/mL for ACTH, 0.25 ng/mL for Cortisol, 0.029 ng/mL for GH, 1.27 pg/mL for GLP1, 0.058 ng/mL for IGF1, 0.056 ng/mL for RLN-3, 0.014 ng/mL for T3, and 0.06 mIU/mL for TSH. The assays demonstrated acceptable precision, with intra-assay coefficients of variation (CV) < 8% and inter-assay CV < 10%.

### 2.4 Sample size estimation

An *a priori* power analysis was conducted using G*Power software (version 3.1; Heinrich-Heine University Düsseldorf, Germany) to estimate the minimum sample size required for detecting group differences in covariance-based analyses. Assuming a medium effect size (f = 0.25), a significance level of α = 0.05, and a statistical power of 0.80, the required minimum sample size was calculated as 90 participants. The final sample consisted of 94 participants (52 patients with OCD and 42 healthy controls), indicating that the study was adequately powered for the planned analyses.

### 2.5 Statistical analysis

All statistical analyses were performed using IBM SPSS Statistics for Windows, Version 26.0 (IBM Corp., Armonk, NY, USA). Descriptive statistics were presented as mean ± standard deviation for continuous variables and as frequencies and percentages for categorical variables.

The distribution of continuous variables was evaluated using the Shapiro–Wilk test together with examination of skewness and kurtosis values. Among the biochemical parameters, ACTH showed a normal distribution. In contrast, cortisol, GH, GLP-1, IGF-1, RLN-3, T3, and TSH did not meet the assumption of normality. Therefore, logarithmic transformation was applied to these variables prior to further analyses. Log-transformed values of the biochemical parameters were used in the covariance-based analyses to meet model assumptions. Group comparisons between the OCD and control groups were performed using the independent samples *t*-test for normally distributed variables and the Mann–Whitney U test for variables that were not normally distributed. Correlation analyses were conducted within the OCD group to examine the associations between biochemical parameter levels and obsessive–compulsive symptom severity as measured by the CY-BOCS. Pearson’s correlation analysis was used for normally distributed variables, whereas Spearman’s rank correlation analysis was used for variables that did not meet the assumption of normality. To evaluate differences in biochemical parameter levels between groups while controlling for potential confounding variables, multivariate analysis of covariance (MANCOVA) and analysis of covariance (ANCOVA) were performed. Age, sex, BMI percentile, total SCARED score, and total CDI score were included as covariates in the models. Given that multiple biochemical parameters were examined simultaneously, multivariate models were preferred to reduce the risk of type I error. When significant group effects were detected, Bonferroni correction was applied for multiple comparisons. A two-tailed *p*-value of < 0.05 was considered statistically significant.

## 3 Results

A total of 94 participants were included in the study, consisting of 52 children with OCD and 42 healthy controls. The mean age was 13.20 ± 2.88 years in the OCD group and 13.02 ± 2.92 years in the control group, and there was no significant difference between the groups (*t* = 0.296, *p* = 0.768). Regarding sex distribution, the OCD group consisted of 31 girls and 21 boys, whereas the control group included 26 girls and 16 boys. There was no significant difference in sex distribution between the groups (χ² = 0.051, *p* = 0.821). Similarly, the BMI percentile did not differ significantly between the groups. The mean BMI percentile was 59.19 ± 31.49 in the OCD group and 68.76 ± 31.22 in the control group (*t* = −1.472, *p* = 0.145). The demographic characteristics of the participants are presented in **Table 1**.

**Table 1:** Demographic and clinical characteristics of the study groups.

Variable	OCD (n = 52)	Controls (n = 42)	Statistic	p
Sex (female/male)	31 / 21 59.6% / 40.4%	26 / 16 61.9% / 38.1%	χ² = 0.051	0.821^a^
Age (years)	13.20 ± 2.88	13.02 ± 2.92	t = 0.296	0.768^b^
BMI percentile	59.19 ± 31.49	68.76 ± 31.22	t = −1.472	0.145^b^
SCARED total	33.83 ± 16.52	18.71 ± 12.35	t = 5.072	<0.001^b^
CDI total	18.87 ± 8.25	8.17 ± 6.75	t = 6.915	<0.001^b^
CY-BOCS obsession	11.00 ± 2.78	–	–	–
CY-BOCS compulsion	10.77 ± 3.06	–	–	–
CY-BOCS total	21.77 ± 5.57	–	–	–

Values are presented as mean ± standard deviation unless otherwise indicated.

BMI, Body mass index; CDI, Children’s Depression Inventory; SCARED, Screen for Child Anxiety Related Emotional Disorders; CY-BOCS, Children’s Yale–Brown Obsessive Compulsive Scale.

^a^Chi-square test.

^b^Student’s t-test.

Children with OCD had significantly higher scores on both the SCARED and the CDI compared with healthy controls. The mean SCARED total score was 33.83 ± 16.52 in the OCD group and 18.71 ± 12.35 in the control group, indicating a significant difference between the groups (*t* = 5.072, *p* < 0.001). Similarly, the mean CDI total score was 18.87 ± 8.25 in the OCD group and 8.17 ± 6.75 in the control group, and this difference was also statistically significant (*t* = 6.915, *p* < 0.001). Detailed results are presented in **Table 1**.

Biochemical parameter levels were compared between the OCD and control groups. ACTH levels did not differ significantly between the groups (*t* = −0.820, *p* = 0.414). For the remaining biochemicals that did not meet the assumption of normality, group comparisons were performed using the Mann–Whitney U test. Cortisol, IGF-1, GLP-1, and T3 levels differed significantly between the groups, with lower levels observed in the OCD group (*z* = −3.651, *p* < 0.001; *z* = −3.168, *p* = 0.002; *z* = −2.019, *p* = 0.043; and *z*= −2.209, *p* = 0.027, respectively). In contrast, GH, RLN-3, and TSH levels did not differ significantly between the groups (*p* = 0.212, *p* = 0.145, and *p* = 0.951, respectively). Detailed descriptive statistics are presented in **Table 2**.

**Table 2:** Comparison of serum ACTH, cortisol, GH, GLP-1, IGF-1, RLN-3, T3, and TSH levels between children with OCD and controls.

Variables	OCD (n = 52) mean ± SD	Controls (n = 42) mean ± SD	OCD (n = 52) median (IQR)	Controls (n = 42) median (IQR)	Statistic	p
ACTH	43.24 ± 21.04	48.15 ± 36.31			t = −0.820^a^	0.414
Cortisol	47.96 ± 21.54	85.58 ± 63.21	44.60 (19.13)	63.85 (54.80)	z = −3.651^b^	<0.001
GH	5.65 ± 3.30	7.32 ± 5.44	4.44 (3.57)	5.39 (4.93)	z = −1.247^b^	0.212
GLP-1	14.25 ± 6.10	21.05 ± 13.75	12.80 (6.53)	15.20 (19.18)	z = −2.019^b^	0.043
IGF-1	76.89 ± 41.11	113.08 ± 59.58	70.60 (44.48)	100.00 (73.08)	z = −3.168^b^	0.002
RLN-3	0.36 ± 0.18	0.53 ± 0.41	0.317 (0.193)	0.390 (0.411)	z = −1.456^b^	0.145
T3	0.25 ± 0.14	0.40 ± 0.31	0.225 (0.127)	0.286 (0.391)	z = −2.209^b^	0.027
TSH	1.05 ± 0.56	1.23 ± 0.96	0.940 (0.567)	0.870 (0.735)	z = −0.061^b^	0.951

ACTH, adrenocorticotropic hormone; GH, growth hormone; GLP-1, glucagon-like peptide-1; IGF-1, insulin-like growth factor-1; RLN3, relaxin-3; T3, triiodothyronine; TSH, thyroid-stimulating hormone, IQR, interquartile range.

^a^Student’s *t*-test.

^b^Mann–Whitney *U* test.

To examine whether the observed group differences remained significant after controlling for potential confounding variables, a multivariate analysis of covariance (MANCOVA) was performed including age, sex, BMI percentile, SCARED total score, and CDI total score as covariates. Because cortisol, GH, GLP-1, IGF-1, RLN-3, T3, and TSH did not meet the assumption of normality, logarithmic transformation was applied to these variables, and the log-transformed values were used in the subsequent covariance-based analyses to satisfy the assumptions of parametric tests. The multivariate model revealed a significant overall effect of group on the combined biochemical parameters (Pillai’s trace = 0.387, *F (8*, 78) = 6.146, *p* < 0.001, ηp² = 0.387). Subsequent univariate ANCOVA analyses were conducted to determine which biochemical parameters contributed to this multivariate effect. To reduce the risk of type I error due to multiple testing, Bonferroni correction was applied. After adjustment for covariates, log-cortisol levels remained significantly different between the groups (*F* (1, 85) = 9.189, *p* = 0.003, ηp² = 0.098). Similarly, significant group effects were observed for log-GLP-1 (*F*(1, 85) = 5.963, *p* = 0.017, ηp² = 0.066), log-IGF-1 (*F*(1, 85) = 6.405, *p* = 0.013, ηp² = 0.070), and log-T3 (*F*(1, 85) = 6.172, *p* = 0.015, ηp² = 0.068). In contrast, ACTH (*F*(1, 85) = 0.186, *p* = 0.668, ηp² = 0.002), log-GH (*F*(1, 85) = 0.566, *p* = 0.454, ηp² = 0.007), and log-TSH (*F*(1, 85) = 0.025, *p* = 0.875, ηp² < 0.001) did not show significant group differences after adjustment. Although log-RLN-3 levels tended to differ between the groups, this effect did not reach statistical significance (*F*(1, 85) = 3.257, *p* = 0.075, ηp² = 0.037). Adjusted mean values from the ANCOVA analyses are presented in **Table 3**.

**Table 3:** Comparison of serum ACTH, cortisol, GH, GLP-1, IGF-1, RLN-3, T3, and TSH levels between children with OCD and controls according to ANCOVA.

Variables	OCD	Controls	F	p	Partial η²
ACTH (pg/mL)	43.87 ± 4.68	47.23 ± 5.78	0.186	0.668	0.002
log Cortisol (ng/mL)	1.65 ± 0.04	1.84 ± 0.05	9.189	0.003	0.098
log GH (ng/mL)	0.72 ± 0.04	0.77 ± 0.05	0.566	0.454	0.007
log GLP-1 (pg/mL)	1.11 ± 0.03	1.25 ± 0.04	5.963	0.017	0.066
log IGF-1 (ng/mL)	1.84 ± 0.04	1.99 ± 0.04	6.405	0.013	0.070
log TSH (mIU/mL)	−0.02 ± 0.04	−0.01 ± 0.05	0.025	0.875	0.000
log T3 (ng/mL)	−0.67 ± 0.04	−0.50 ± 0.05	6.172	0.015	0.068
log RLN-3 (ng/mL)	−0.50 ± 0.04	−0.38 ± 0.05	3.257	0.075	0.037

ACTH, adrenocorticotropic hormone; GH, growth hormone; GLP-1, glucagon-like peptide-1; IGF-1, insulin-like growth factor-1; RLN3, relaxin-3; T3, triiodothyronine; TSH, thyroid-stimulating hormone.

Covariates: age, sex, BMI percentile, SCARED total score, and CDI total score.

Log: logarithmic transformation applied prior to analysis.

Bonferroni correction was applied for multiple comparisons.

Correlation analyses were conducted within the OCD group to examine the associations between biochemical parameters and symptom severity as measured by the CY-BOCS. Pearson correlation analysis was used for ACTH, while Spearman’s rank correlation analysis was applied for the remaining biochemical parameters. No significant correlations were found between ACTH levels and CY-BOCS total, obsession, or compulsion scores (*p* > 0.05 for all). Similarly, GH, GLP-1, IGF-1, RLN-3, T3, and TSH levels were not significantly associated with CY-BOCS scores (*p* > 0.05). A weak negative correlation was observed between cortisol levels and the CY-BOCS compulsion subscale (r = −0.281, *p* = 0.044). However, cortisol levels were not significantly associated with the obsession subscale or total CY-BOCS scores (*p* > 0.05). Correlation analyses did not reveal any significant associations between serum biochemical parameter levels and CY-BOCS scores after Bonferroni correction for multiple comparisons. Detailed correlation coefficients are presented in **Table 4**.

**Table 4:** Correlations between serum ACTH, cortisol, GH, GLP-1, IGF-1, RLN-3, T3, and TSH levels and CY-BOCS scores in the OCD group.

Variables	CY-BOCS Compulsion	CY-BOCS Obsession	CY-BOCS total
ACTH	r = 0.024^a^	r = 0.105^a^	r = 0.066^a^
Cortisol	r = −0.281^b^	r = −0.164^b^	r = −0.237^b^
GH	r = 0.061^b^	r = 0.142^b^	r = 0.093^b^
GLP-1	r = 0.083^b^	r = 0.144^b^	r = 0.134^b^
IGF-1	r = −0.008^b^	r = 0.042^b^	r = 0.016^b^
RLN-3	r = 0.014^b^	r = 0.082^b^	r = 0.048^b^
T3	r = −0.043^b^	r = 0.046^b^	r = −0.010^b^
TSH	r = −0.083^b^	r = 0.028^b^	r = −0.026^b^

ACTH, adrenocorticotropic hormone; GH, growth hormone; GLP-1, glucagon-like peptide-1; IGF-1, insulin-like growth factor-1; RLN3, relaxin-3; T3, triiodothyronine; TSH, thyroid-stimulating hormone; CY-BOCS, Children’s Yale-Brown Obsessive Compulsive Scale.

^a^Pearson correlation analysis.

^b^Spearman correlation analysis.

Bonferroni-corrected significance threshold: p < 0.002.

## 4 Discussion

In this medication-free sample of children and adolescents with OCD, significant group differences were observed in several biochemical parameters after controlling for potential confounding variables, including age, sex, BMI percentile, and levels of anxiety and depressive symptoms. Specifically, serum cortisol, IGF-1, GLP-1, and T3 levels were significantly lower in the OCD group compared with healthy controls, whereas ACTH, GH, TSH, and RLN-3 levels did not differ significantly between groups. Correlation analyses further indicated that biochemical parameter levels were generally not associated with OCD severity. Rather than reflecting isolated abnormalities in single hormonal pathways, the present findings may indicate a multi-system neuroendocrine dysregulation involving stress-related, metabolic, neurodevelopmental, and thyroid-related signaling pathways in pediatric OCD.

Our findings indicate that serum cortisol levels were significantly lower in children and adolescents with OCD compared with healthy controls, whereas ACTH levels did not differ significantly between groups. Previous findings on HPA axis activity in pediatric OCD have been inconsistent. For example, Bilgiç et al. reported significantly elevated ACTH levels but no significant difference in cortisol levels in medication-free children with OCD (8), whereas Şimşek et al. found both ACTH and cortisol levels to be elevated in pediatric OCD (17). Findings in adult samples have also been heterogeneous, although several studies have reported increased cortisol levels in patients with OCD (9). In contrast, Koumantarou Malisiova et al. reported lower hair cortisol concentrations in adults with OCD, suggesting that prolonged stress exposure may be associated with chronic downregulation of the HPA axis (52). Taken together, these discrepant findings may be related to methodological differences across studies, including age group, medication status, biological sampling methods, and timing of assessment. They may also indicate that HPA axis functioning in OCD is not characterized by a uniform pattern of activation, but rather by a more complex dysregulation that may vary across developmental stages and different phases of disorder. In addition, salivary cortisol measurements may provide complementary information regarding the biologically active free fraction of cortisol, diurnal variation, and dynamic HPA axis regulation. Future longitudinal studies incorporating salivary cortisol assessments may help to better clarify HPA axis alterations in pediatric OCD.

The present findings showed that serum RLN-3 levels did not differ significantly between children and adolescents with OCD and healthy controls, although a trend-level difference was observed. To our knowledge, no previous study has directly examined RLN-3 levels in OCD. While research in this area remains very limited, Ali et al. reported reduced serum RLN-3 levels in patients with major depressive disorder (53), whereas Erden et al. found higher RLN-3 levels in children with autism spectrum disorder (21). These findings suggest that RLN-3 may be involved in neuropsychiatric and neurodevelopmental processes, although its direction of change may vary across disorders. In this context, our findings do not point to a clear relationship between RLN-3 and pediatric OCD, although a potential contribution may still exist. Considering the role of RLN-3 in the modulation of CRH neurons and stress-related circuits (18), future studies may further clarify whether this neuropeptide system contributes to compulsive symptom domains. Further research in larger samples is warranted to better elucidate the possible role of RLN-3 in the neurobiology of OCD.

We observed significantly lower serum GLP-1 levels in children and adolescents with OCD compared with healthy controls. To our knowledge, no previous study has directly examined GLP-1 levels in OCD. Although GLP-1 is primarily recognized as a metabolic hormone involved in glucose regulation and appetite control, growing evidence suggests that it also exerts important effects within the central nervous system (22, 23, 26). Detka and Głombik emphasized that GLP-1 signaling may be involved in neuroprotection, synaptic plasticity, neuroinflammation, and stress regulation (54), while De Giorgi and colleagues highlighted its potential role in reward-related, cognitive, and neuroimmune pathways. However, findings in the broader psychiatric literature remain inconsistent (55). In addition, Järvinen and colleagues reported reduced compulsive food-related thoughts and repetitive behaviors following liraglutide treatment in an adolescent with autism (27). Taken together, these findings suggest that GLP-1 signaling may be relevant to compulsive and repetitive symptom domains. Therefore, the lower GLP-1 levels observed in our pediatric OCD sample may indicate a potential involvement of GLP-1 signaling in the neurobiology of OCD, which warrants further investigation in future studies.

Similarly, serum IGF-1 levels were significantly lower in children and adolescents with OCD compared with healthy controls, whereas GH levels did not differ significantly between the groups. Previous research examining the somatotropic axis in OCD has been limited and has largely focused on adult populations. For example, Brambilla et al. reported that although basal GH levels did not differ between patients with OCD and healthy controls, GH responses to growth hormone-releasing hormone stimulation were significantly blunted in patients, suggesting dysregulation in the regulatory mechanisms controlling GH secretion (39). Similarly, Kluge et al. demonstrated altered nocturnal GH secretion patterns in adults with OCD, characterized by a reduced sleep-onset GH peak and a more fragmented secretion pattern (38). Research regarding IGF-1 in OCD is even more limited. In a case–control study conducted in adults, Rosso et al. reported significantly higher serum IGF-1 levels in drug-naïve OCD patients compared with healthy controls, suggesting that alterations in growth-related neurotrophic signaling may be involved in the pathophysiology of the disorder (37). In contrast to this finding, our results indicated lower IGF-1 levels in pediatric OCD patients. This discrepancy may be related to differences in age groups, developmental stage, or methodological approaches across studies. To our knowledge, studies investigating GH and IGF-1 levels specifically in children and adolescents with OCD are still scarce. Considering the fundamental role of the somatotropic axis in brain maturation and neurodevelopmental processes, further research is warranted to clarify the potential contribution of GH–IGF-1 signaling to the neurobiology of pediatric OCD.

Our results also showed significantly lower serum T3 levels in children and adolescents with OCD compared with healthy controls, whereas TSH levels did not differ significantly between groups. Previous studies investigating thyroid-related changes in OCD have yielded inconsistent results. While some studies have reported reduced T3 levels (44), others, including pediatric samples, have found elevated T3 levels (43). Likewise, findings for TSH have been mixed, ranging from normal basal levels (44) to a blunted TSH response (56). Taken together, the available literature does not indicate a consistent pattern of thyroid dysfunction in OCD. In this context, the lower T3 levels observed in our study, in the absence of a significant difference in TSH, may suggest that thyroid-related changes in pediatric OCD are more likely to be reflected at the level of peripheral hormone activity rather than pituitary regulation. However, the clinical significance of this finding remains unclear, and further studies are needed to clarify the possible role of thyroid-related mechanisms in the neurobiology of pediatric OCD.

Several limitations of the present study should be considered. First, the cross-sectional design precludes any causal interpretation of the observed associations. Second, although the sample size was adequate according to the *a priori* power analysis, replication in larger samples is still needed. Third, all biochemical measurements were based on peripheral serum levels obtained at a single time point, which may not fully reflect dynamic neuroendocrine activity or central nervous system processes. Fourth, serum-based analyses were preferred in the present study because multiple neuroendocrine parameters were simultaneously evaluated from fasting morning blood samples obtained under standardized laboratory conditions. However, serum cortisol measurements may not fully capture diurnal cortisol fluctuations and dynamic HPA axis regulation compared with salivary cortisol assessments.

Despite these limitations, the present study also has several important strengths. To our knowledge, this is one of the few studies to simultaneously examine multiple neuroendocrine markers related to stress regulation, growth processes, metabolic signaling, and thyroid function in pediatric OCD. The inclusion of a medication-free sample and the exclusion of major psychiatric and medical comorbidities increased the clinical homogeneity of the patient group and reduced potential confounding effects. In addition, the use of standardized diagnostic interviews and validated symptom measures strengthened the clinical characterization of the sample. Another notable strength is that the analyses were performed both at the univariate level and with adjustment for relevant covariates, allowing a more robust evaluation of group differences. Taken together, these features enhance the reliability of the findings and contribute to the growing body of research investigating the biological underpinnings of pediatric OCD. Future studies integrating longitudinal designs and multimodal biological measurements may further clarify how these neuroendocrine alterations contribute to the development and course of OCD.

## 5 Conclusion

In conclusion, the present findings suggest that pediatric OCD may be associated with alterations in several neuroendocrine pathways, including cortisol, IGF-1, GLP-1, and T3. These findings contribute to the growing literature on the potential involvement of neuroendocrine mechanisms in the pathophysiology of OCD during development. However, further longitudinal and experimental studies are needed to better clarify the underlying biological mechanisms and the clinical significance of these findings. These findings also support the hypothesis that pediatric OCD may involve dysregulation across multiple interacting neuroendocrine pathways rather than alterations within a single hormonal system.

## Data Availability

The raw data supporting the conclusions of this article will be made available by the authors, without undue reservation.
